# Treating Steroid Refractory Intestinal Acute Graft-vs.-Host Disease With Fecal Microbiota Transplantation: A Pilot Study

**DOI:** 10.3389/fimmu.2018.02195

**Published:** 2018-09-25

**Authors:** Xiaofei Qi, Xuewei Li, Ye Zhao, Xiaojin Wu, Feng Chen, Xiao Ma, Faming Zhang, Depei Wu

**Affiliations:** ^1^Department of Hematology, The First Affiliated Hospital of Soochow University, Suzhou, China; ^2^Medical Center for Digestive Diseases, The Second Affiliated Hospital of Nanjing Medical University, Nanjing, China

**Keywords:** fecal microbiota transplantations, refractory gastrointestinal, graft-vs.-host disease, diarrhea, a pilot study

## Abstract

Patients with steroid refractory gastrointestinal (GI) tract graft- vs.-host disease (GvHD) face a poor prognosis and limited therapeutic options. To accurately assess the efficacy and safety of fecal microbiota transplantation (FMT) in treating steroid refractory GI tract GvHD, we conducted a pilot study involving eight patients. Having received FMTs, all patients' clinical symptoms relieved, bacteria enriched, and microbiota composition reconstructed. Compared to those who did not receive FMT, these eight patients achieved a higher progression-free survival. FMT can serve as a therapeutic option for GI tract aGVHD, but its effectiveness and safety need further evaluations.

**Clinical Trial Registration:** NCT03148743.

## Introduction

Hematopoietic stem cell transplantation (HSCT) is a potential curative strategy for patients with hematological malignancy and its complications, including infections, multi-organ failure, and graft-versus-host disease (GvHD) (1, 2). GvHD, especially acute GvHD (aGvHD), is a major cause of morbidity and mortality. Gut-GvHD-related complications also appear as the most common causes of post-transplantation death (1). Apart from abdominal pain, satiety, anorexia, nausea, and vomiting, diarrhea is the most significant symptom of GvHD and the most expected risk of gut GvHD-related mortality (3, 4). Currently, the first-line therapy for GvHD is use of glucocorticoids, but almost half of the patients do not respond efficiently (5). By far, no second-line treatment has been established. Therefore, effective treatments are urgently needed to treat patients with steroid refractory aGvHD (6).

Recent studies have verified the changed intestinal diversity in patients with GvHD. Through evaluating the entire spectrum of aGvHD, these studies corroborated that gut microbiota may modulate systemic alloimmune responses (7–11). Under a normal physiological condition, microbes of highly diversity in the human GI tract participate in intestinal inflammation and immune responses (12). However, after allo-HSCT, the abnormal gut microbiota damages GI mucosa, and eventually collapses the intestinal microbiota diversity (10, 11).

Fecal microbiota transplantation (FMT), a therapy that restores microbiota system through infusing a fecal suspension from a healthy donor into a receiver's GI tract (13), has been proven effective for recurrent Clostridium difficile infection (14). Scientists have used this therapy to cure other human diseases (such as inflammatory bowel disease). Considering the poor prognosis of steroid refractory GI tract aGvHD and limited therapeutic options, we speculated that FMT might be effective to cope with these challenges. Therefore, we conducted this pilot study to evaluate the effectiveness of FMT in treating steroid refractory GI tract aGvHD.

## Patients and methods

### Study design and patient eligibility

Eight patients with steroid refractory GI tract aGvHD were enrolled in the study. Protocols were approved by the Institutional Review Board of the First Affiliated Hospital of Soochow University. All patients had been informed the possible adverse reactions and had signed written informed consent to receive FMT voluntarily. The study was registered with ClinicalTrials.gov as #NCT03148743. The severity of GI aGVHD was graded according to the Center for International Blood and Marrow Transplant Research (CIBMTR) criteria (15). The patients were treated with standard first-line treatment with corticosteroids and at least 1 s-line therapy. They were diagnosed with steroid refractory gut aGVHD if any of the following characteristics presented: (a) progression of acute GvHD or lack of response to a treatment using 2 mg/kg per day or more methylprednisolone for at least 72 h; (b) progression of GI tract aGvHD even after a treatment using 2 mg/kg per day or additional methylprednisolone for at least 48 h; (c) progression of GI tract aGvHD to stage 4 after 24 h of daily 2 mg/kg or additional methylprednisolone (16).

Patients with uncontrollable infection, irreversible organ failure, and other abnormal conditions that might interfere with informed consent or evaluation were excluded. Efficacy endpoints were defined when aGvHD overall grade, organ response, or overall survival (OS) showed a turning point. For each patient, the safety evaluation stopped at 90 days after the first FMT, or any moment when the patient died or quitted.

### FMT procedures

Frozen fecal microbiota was obtained from China fmtBank (Nanjing, China). The two donors included in our research were females aged 23 years. Forty to fifty milliliters frozen fecal microbiota were suspended in 200 ml of warm normal saline every time and delivered into intestine within 2–4 min through a nasoduodenal tube.

### Efficacy and safety assessment

FMT efficacy and safety were evaluated according to the severity of symptoms such as abdominal pain, diarrhea (frequency and volume), and bloody purulent stool in 2 weeks. Clinical remission was defined if diarrhea and intestinal spasms and/or bleeding disappeared, or stool volume decreased by ≥500 mL on average in 3 days. Clinical improvement was defined if the stool volume decreased by < 500 mL, and the enterosepsis and bleeding relieved. Safety was evaluated according to adverse events during FMT and follow-up. Other events, such as death, GvHD of other organs, active infection of CMV, or EB virus within 90 days after the diagnosis of steroid refractory GI tract GvHD, were also recorded. Progression-free survival (PFS) was defined as no progress with GI-GvHD, no death, no other organs appeared GVHD, no new infection with CMV and EBV during these 90 days. Period from GI-GVHD was diagnosed to July 2017 was as the statistical time of OS.

### Stool sample collection and microbial community analysis

Fecal samples from patients and donors were collected and stored at −80°C. Then, 16s rRNA sequencing was used for microbiota analysis. The phylum- and family-level analyses were used to show the composition of fecal bacteria. Moreover, the Shannon diversity index was used to depict the diversity of microbiota (17).

### Statistical analysis

Data were analyzed using SPSS 16.0. Comparison between the two groups was conducted using Student's *t*-test. Comparison between PFS and OS rates was presented in a Kaplan–Meier curve. *P* < 0.05 was considered statistically significant.

## Results

### Patients characteristics

**Figure 1:**
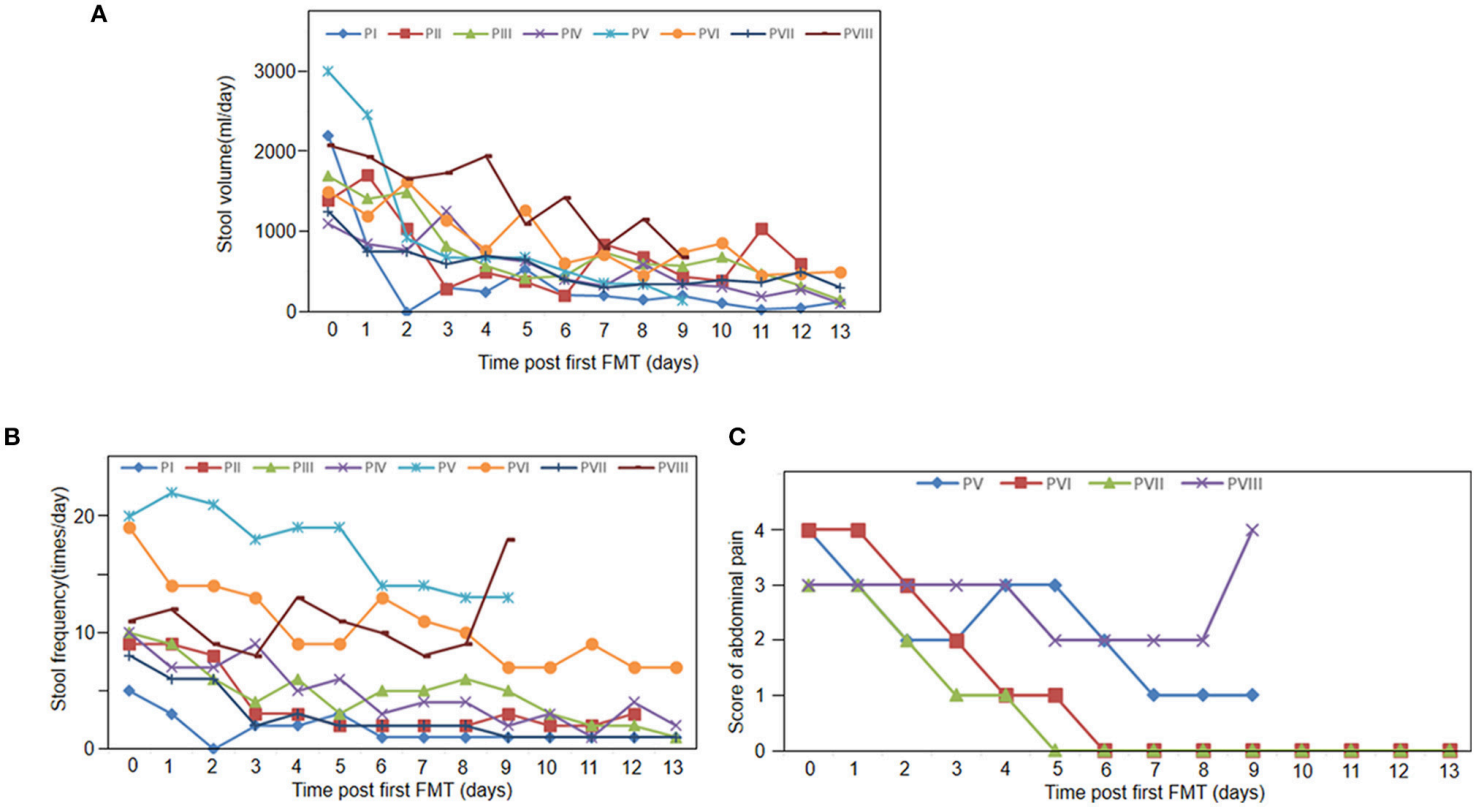
Clinical response to FMT. **(A)** Stool volumes of all 8 patients pre FMT and 13 days post FMT. **(B)** Stool frequency of all 8 patients pre FMT and 13 days post FMT. **(C)** Abdominal pain score at baseline and 13 days follow-up after FMT (*n* = 4).

Eight patients with steroid refractory GI tract aGvHD were enrolled in this study (mean age of 35.6 years, ranging 20–48 years; male/female ratio, 3/5; Table 1). *Clostridium difficile* infection was not observed among all patients. The mean time of the onset of GI tract aGvHD (days after allo-HSCT) was +79.7, ranging from +21 to +369 (Table 1). Immunosuppressant were given to all patients (Table 2). All eight patients were diagnosed with GI tract GvHD, including two with combined liver GvHD and five with skin GvHD (Table 3). The mean stool volume of all patients was 1,777 mL/day, ranging from 1,100 to 3,000 mL/day. The mean stool frequency was 11.5 times/day, ranging from 5 to 20 times/day (Table 4, Figures 1A,B).

**Table 1:** Clinical characteristics of patients treated with FMT.

	**Range of age**	**Date of allo-HSCT**	**Hematologic disease**	**Donor gender, relationship**	**Stem cell source**	**Number of CD34^+^ x10^6^ content per kg recipient body weight**	**GvHD prophylaxis**	**Onset of aGvHD (days after alloHSCT; organ, stage)**	**Onset of GI-aGvHD (days after allo-HSCT)**
PI	36–40	2016/9/1	CML	Male MSD	BM	4.7	ATG+CsA+MMF +short-term MTX	+74; GI,IV	+74
PII	40–45	2016/12/1	AML	Female Haplo-HSCT	cord blood+BM	3.52	ATG+CsA+MMF +short-term MTX	+20; liver,I	+28
PIII	26–30	2017/3/3	ALL	Male Haplo-HSCT	BM	2.346+4.74	ATG+CsA+MMF +short-term MTX	+21; GI,IV	+21
PIV	30–35	2017/3/1	MDS	Male Haplo-HSCT	BM	2.12+2.21	ATG+CsA+MMF +short-term MTX	+23; skin,I	+33
PV	46–50	2017/2/28	MDS	Female MSD	PBSC	2.583	ATG+CsA+MMF +short-term MTX	+25; skin,I	+53
PVI	40–45	2017/5/9	HAL	Male Haplo-HSCT	BM+PBSC	2.38	ATG+CsA+MMF +short-term MTX	+53d; GI,IV	+27
PVII	Female, 28, Mongolia	2017/5/6	ALL	Female Haplo-HSCT	cord blood+BM	4.97	ATG+CsA+MMF +short-term MTX	+30; skin,I	+33
PVIII	Male, 20, Mongolia	2016/6/13	AML	UD-HSCT	PBSC	4.58	ATG+CsA+MMF +short-term MTX	+357; skin,I	+369

*AML, acute myeloid leukemia; ALL, acute lymphoblastic leukemia; HAL, hybrid acute leukemia; CML, chronic myelocytic leukemia; MDS, myelodysplastic syndromes; PBSC, peripheral blood stem cells; BM, bone marrow stem cell; ,haplo-HSCT, haploid allogeneic hematopoietic stem cell transplantation; ATG, anti-thymocyte globulin; CsA, cyclosporine; MMF, mycophenolate mofetil; MTX, methotrexate*.

**Table 2:** Immunosuppressants given to all patient with introduction of FMTs.

	**Methylprednisolone**	**Budesonide**	**Etanercept**	**Basiliximab**	**Tacrolimus**	**Cyclophosphamide**	**Cyclosporin**
PI	+	–	–	+	+	+	–
PII	+	+	–	+	+	+	+
PIII	+	+	+	+	+	–	–
PIV	+	+	+	+	+	+	+
PV	+	–	–	+	+	+	–
PVI	+	–	–	+	+	–	+
PVII	+	–	–	+	+	+	–
PVIII	+	–	–	–	+	+	–

**Table 3:** Factor related FMT in patients with FMT treated.

	**Skin (Stage)**	**Liver (Stage)**	**GI (Stage)**	***Clostridium difficile***	**Times of FMTs**	**Time point of first/last FMT (days after allo-HSCT)**	**FMT donor relationship**
PI	–	–	IV	–	2	+84; +92	Unrelated donor
PII	–	I	IV	–	2	+38; +42	Unrelated donor
PIII	–	–	IV	–	1	+35	Unrelated donor
PIV	I	II	IV	–	2	+50; +56	Unrelated donor
PV	–	–	IV	–	1	+70	Unrelated donor
PVI	I	–	IV	–	2	+35; +42	Unrelated donor
PVII	I	–	IV	–	1	+52	Unrelated donor
PVIII	I	–	IV	–	1	+375	Unrelated donor

**Table 4:** Clinical response to FMT.

**Stool volume (ml/day)/stool frequency (times/day)**				**Infectious events (a week after FMT)**	**GI-aGvHD clinical outcome**	**Overall outcome**
	**Day 0 after first FMT**	**Day 3 after first FMT**	**Day 13 after first FMT**			
PI	2,200/5	300/2	50/1	Without	Cure	Alive
PII	1,395/9	290/3	600/3 (day 12)	Without	Relapse	Died
PIII	1,695/10	820/4	150/1	Without	Remission	Died
PIV	1,100/10	1,255/9	100/2	Without	Cure	Alive
PV	3,000/20	680/18	140/13 (day 9)	Without	Improvement	Died
PVI	1,500/19	770/9	500/7	Without	Cure	Alive
PVII	1,250/8	600/2	300/1	Without	Cure	Alive
PVIII	2,080/11	1,735/8	680/18 (day 9)	Without	Relapse	Died

### Case serials and FMT outcomes

Table 4 exhibits the clinical efficacy of FMT. FMT was applied in four patients once and four patients twice (Table 3). In the subsequent follow-up, because of the recurrent or new emergent complications of GvHD, two patients gave up the treatment and quickly died (at day 12 and 50 after FMT, respectively). One patient died of cerebral hemorrhage caused by thrombocytopenia (at day 9 after FMT). In addition, due to economic reasons, one patient gave up the treatment at day 9 after FMT and died (Table 4).

Figure 1 shows that all the patients achieved clinical symptomatic remission after the first FMT. Among patients with diarrhea, their stool volumes and frequencies were reduced to 3–4 times/day after FMT (Figures 1A,B). However, diarrhea relapsed to one patient at day 11 (patient II). One week after the first FMT, abdominal pain was also decreased. At day 9, abdominal pain and stool frequency of patient VIII increased (Figures 1A,C). In the 2-week follow-up after the first FMT, two patients' diarrhea and all the other five patients' abdominal pain disappeared (the left three died; Table 4).

### Fecal microbiota analysis

The change of intestinal microbiota was analyzed with fecal samples from six patients (before FMT and a week after the first FMT). Lacking 16s sequencing data of two patients, the change in the two patients could not be evaluated.

**Figure 2:**
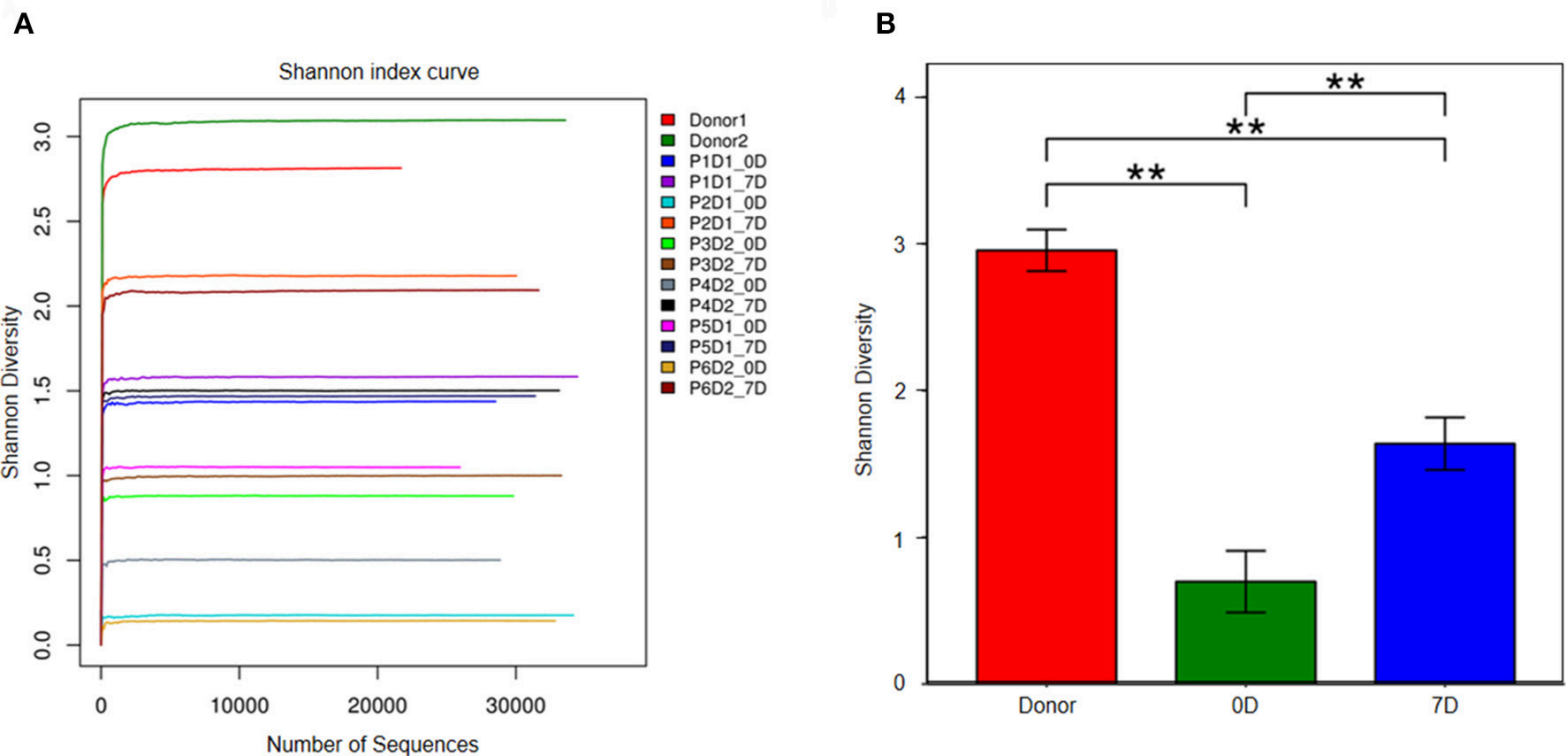
Analysis of fecal microbiota in donor and 6 patients. **(A)** The diversity of fecal microbiota in all sample (Shannon's diversity index). Px means patient number, Dx means donor number, xD means day after FMT. **(B)** Shannon's diversity index change in donor group, pre-FMT and post-FMT samples. **p < 0.01.

**Figure 3:**
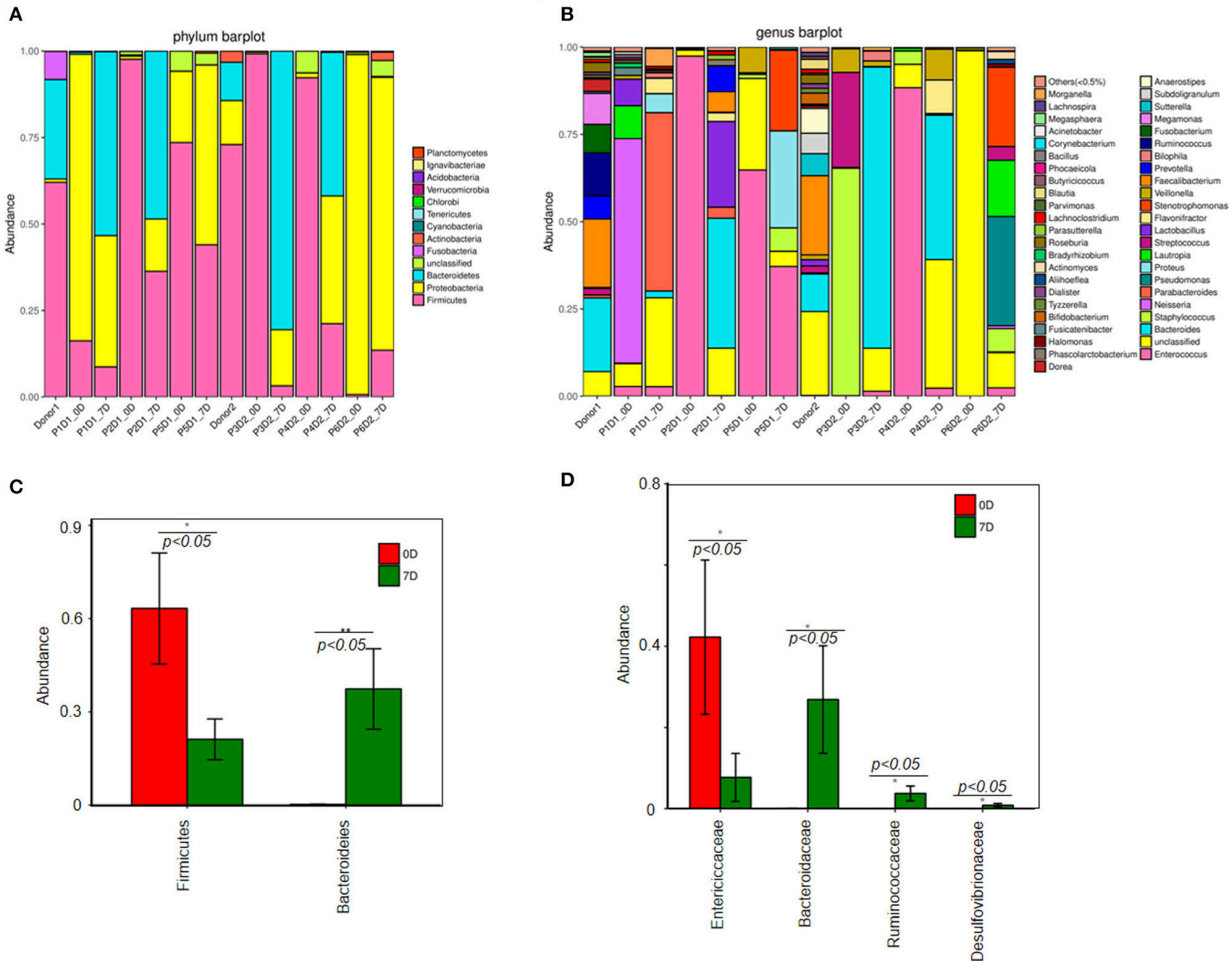
Change of fecal microbiota before and after FMT. **(A)** Analysis of fecal microbiota composition in all samples at the phylum level. **(B)** Analysis of fecal microbiota composition in all samples at the genus level. Difference of fecal microbiota composition between pre-FMT and post-FMT at the phylum level **(C)**. **(D)** Genus level.

Figure 2 illustrates that prior to FMT the diversity of fecal microbiota in fecal samples of the patients decreased compared with that of the donors (Figures 2A,B). At the phylum level, *Firmicutes, Bacteroidetes*, and *Proteobacteria* were three major bacteria found in the fecal microbiota of the donors. However, the ratio of *Firmicutes* to *Proteobacteria* got imbalanced in the fecal microbiota of patients with steroid refractory GI tract GvHD, and ratio of *Bacteroidetes* significantly decreased (Figure 3A).

The results of 16s rRNA gene sequencing also confirmed that the bacterial diversity improved 1 week after FMT (Figure 2B). Similar to these results, the microbiota composition was reconstructed, showing a trend back to normal (Figures 3A,B). Moreover, pre-FMT samples had a significantly high level of *Firmicutes* (Figure 3A) and *Enterococcaceae* (Figure 3B). Post-FMT samples had a significantly high level of *Bacteroidetes* (Figure 3C) and *Bacteroidaceae, Ruminococcaceae* and *Desulfovibrionaceae* (Figure 3D). Beneficial bacteria, such as *Bacteroides*, were dominant in these cases after FMTs (Figure 3C).

### Efficacy and safety of FMT

**Figure 4:**
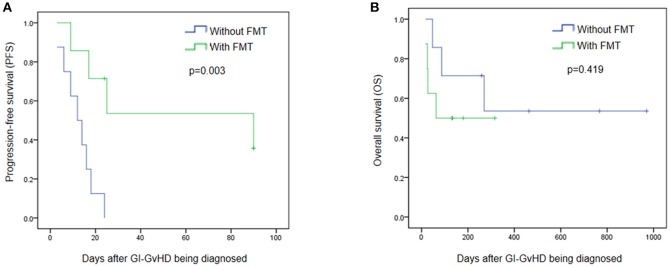
Efficacy and safety of FMT. **(A)** Progression free survival (PFS) between patients treated with FMT and without FMT in 90 days after the steroid refractory GI-GvHD being diagnosed. **(B)** Overall survival (OS) in the refractory and steroid- insensitive GI-GvHD patients who treated with FMT/without FMT were shown. Days means day after the steroid refractory GI-GvHD being diagnosed.

No severe adverse events were observed during and after FMT. To further clarify the safety and efficacy of FMT, we simultaneously conducted a retrospective study with another eight patients who had been diagnosed with steroid refractory GI tract GvHD but not received FMT in our center (Supplement Table 1). Figure 4 exhibits that, within 90 days after the diagnosis of refractory and steroid-insensitive GI tract GvHD, progression-free survival (PFS) improved in the FMT group (*p* = 0.003; Figure 4A), OS showed no difference between the two groups (*p* = 0.419) during the period from GI-GVHD was diagnosed to July 2017 (Figure 4B).

## Discussion

Patients with steroid refractory GI tract GvHD face a high mortality and limited therapeutic options (6). Recent data found that loss of intestinal bacterial diversity in GvHD, manifested with the enriching *Enterococci*, was also observed in GI-aGVHD patients after allo-HSCT (9) and the microbial metabolites in this condition can directly influence target tissues (13, 18).

Fecal microbiota transplantation (FMT) is a process of transferring normal fecal microorganisms into a patient's GI tract to restore his/her microbiota (13). In our pilot study, all the eight patients diagnosed with steroid refractory GI tract GvHD were given FMT when immunosuppressive therapy and other novel treatments failed. Consequently, relieved clinical symptoms were observed among all patients (e.g., decreased stool volume and frequency and milder abdominal pain), which may be associated with the increased microbial richness. The microbiota composition was also restored after FMTs. After FMT, diversity of microbiota of the patients neared that of the donors. Beneficial bacteria were dominant after FMT.

In our research, we did not observe any adverse event, such as hypoxia, paroxysmal atrial fibrillation (PAF), transplant-associated lower gastrointestinal bleeding, cholestatic liver damage. One patient experienced thrombocytopenia after FMT, an event that could not be completely ruled out. During FMT, we temporarily suspended the prophylactic antibiotics to protect the transplanted microbiome, and did not observe any immediate procedure-related infections. In the FMT group, PFS kept improving during the 90 days' follow-up. OS showed no significant difference during the observation period between the groups with and without FMT. However, to more accurately assess the efficacy and safety of FMT in treating steroid refractory GI tract GvHD, large-sample research is needed.

In summary, the diversity of intestinal microbiota can be undermined by allo-HSCT. Previous findings (10, 11) and our present data corroborate that interventions should be taken to simultaneously maintain intestinal diversity and improve outcomes of allo-HSCT. FMT can serve as a therapeutic option for GI tract aGVHD, but its effectiveness and safety need further evaluations.

## Author contributions

XQ and DW contributed to the study concept and design. XL, YZ, XW, FC, and XM collected the clinical samples. XQ and FZ performed the experiments. XQ, XL, and YZ performed bioinformatics analyses. XQ, XL, and YZ wrote the manuscript. DW supervised the study.

### Conflict of interest statement

The authors declare that the research was conducted in the absence of any commercial or financial relationships that could be construed as a potential conflict of interest.
