# Supplementary material for: Treating Steroid Refractory Intestinal Acute Graft-vs.-Host Disease With Fecal Microbiota Transplantation: A Pilot Study
Source: Front Immunol. 2018 Sep 25;9:2195. doi: 10.3389/fimmu.2018.02195 (PMC6167440; doi:10.3389/fimmu.2018.02195)
Supplement: Supplementary file 1 [file Table_1.DOCX]

Supplement table 1. Clinical characteristics of eight refractory and steroid- insensitive patients without FMT treated

|  | **patient**  **cI** | **Patient**  **cII** | **Patient**  **cIII** | **Patient**  **cIV** | **Patient**  **cV** | **Patient**  **cVI** | **patient**  **cVII** | **patient cVIII** |
| --- | --- | --- | --- | --- | --- | --- | --- | --- |
| **Gender, age, ethnicity** | male,57, Mongolia | male,10,  Mongolia | male,27,  Mongolia | female,29,  Mongolia | male,33,  Mongolia | male,28, Mongolia | male,17,  Mongolia | female,29,  Mongolia |
| **Date of allo-HSCT** | 2016/12/28 | 2016/7/19 | 2016/8/15 | 2016/6/3 | 2015/7/28 | 2015/12/17 | 2015/8/19 | 2014/12/27 |
| **Hematologic disease** | AML | HAL | CML | AA | AML | MDS | AA | LBL/ALL |
| **Donor gender, relationship** | female Haplo-  HSCT | female Haplo-  HSCT | male Haplo-  HSCT | male  Haplo-  HSCT | male Haplo-  HSCT | male Haplo-  HSCT | male Haplo-  HSCT | female Haplo-  HSCT |
| **Stem cell source** | BM+  PBSC | BM+  PBSC | BM+  PBSC | Cord blood+BM | BM+  PBSC | BM+  PBSC | BM+  PBSC | PBSC |
| **Number of CD34^+^ x10^6^ content /kg recipient body weight** | 3.50 | 2.598 | 4.07 | 4.64 | 4.02 | 1.69 | 5.0 | 2.66 |
| **GvHD prophylaxis** | ATG+CsA+MMF +short-term MTX | ATG+CsA+MMF +short-term MTX | ATG+CsA+MMF +short-term MTX | ATG+CsA+MMF +short-term MTX | ATG+CsA+MMF +short-term MTX | ATG+CsA+MMF +short-term MTX | ATG+CsA+MMF +short-term MTX | ATG+CsA+MMF +short-term MTX |
| **Onset of aGvHD (days after alloHSCT;organ, stage)** | +32; GI-GVHD IV | +33; GI-GVHD IV | +33; skin-GVHD I | +25d; skin-GVHDⅠ | +28; GI-GVHD IV | +32; GI-GVHD IV | +23; skin-GVHDⅠ | +19d; skin-GVHDⅠ |
| **Onset of GI-aGvHD (days after allo-HSCT)** | +32 | +33 | +38 | +32 | +28 | +32 | +30 | +20 |
| **aGvHD therapy** |  |  |  |  |  |  |  |  |
| Methylprednisolone | **+** | **+** | **+** | **+** | **+** | **+** | **+** | **+** |
| Budesonide | **-** | **-** | **+** | **-** | **-** | **-** | **+** | **+** |
| Etanercept | **+** | **-** | **+** | **-** | **-** | **-** | **+** | **+** |
| Basiliximab | **+** | **+** | **+** | **+** | **+** | **+** | **+** | **+** |
| Tacrolimus | **+** | **+** | **+** | **+** | **+** | **+** | **+** | **+** |
| Cyclophosphamide | **+** | **+** | **+** | **-** | **+** | **+** | **+** | **+** |
| Cyclosporin | **+** | **-** | **-** | **-** | **+** | **-** | **-** | **-** |
| **Max stool volume(ml/day)** | 1900 | 1400 | 1600 | 1420 | 2390 | 3600 | 3700 | 1450 |
| **GI-aGvHD clinical outcome** | cure | improvement | improvement | remission | remission | improvement | cure | cure |
| **Overall outcome** | alive | died | died | alive | lost | died | alive | alive |

AML (acute myeloid leukemia),ALL (acute lymphoblastic leukemia),HAL (hybrid acute leukemia),CML (chronic myelocytic leukemia),AA (aplastic anemia),LBL/ALL (lymphoblastic lymphoma/ acute lymphoblastic leukemia),PBSC (Peripheral Blood Stem Cells),BM (bone marrow stem cell),haplo-HSCT(Haploid Allogeneic Hematopoietic Stem Cell Transplantation).

ATG (anti-thymocyte globulin),CsA (cyclosporin),MMF (mycophenolate mofetil),MTX (methotrexate)
